# Human cancer cells utilize mitotic DNA synthesis to resist replication stress at telomeres regardless of their telomere maintenance mechanism

**DOI:** 10.18632/oncotarget.24745

**Published:** 2018-03-23

**Authors:** Özgün Özer, Rahul Bhowmick, Ying Liu, Ian D. Hickson

**Affiliations:** ^1^ Center for Chromosome Stability and Center for Healthy Aging, Department of Cellular and Molecular Medicine, University of Copenhagen, 2200 Copenhagen, Denmark

**Keywords:** alternative lengthening of telomeres, common fragile sites, RAD52, MiDAS, MUS81, Chromosome

## Abstract

Telomeres resemble common fragile sites (CFSs) in that they are difficult-to-replicate and exhibit fragility in mitosis in response to DNA replication stress. At CFSs, this fragility is associated with a delay in the completion of DNA replication until early mitosis, whereupon cells are proposed to switch to a RAD52-dependent form of break-induced replication. Here, we show that this mitotic DNA synthesis (MiDAS) is also a feature of human telomeres. Telomeric MiDAS is not restricted to those telomeres displaying overt fragility, and is a feature of a wide range of cell lines irrespective of whether their telomeres are maintained by telomerase or by the alternative lengthening of telomeres (ALT) mechanism. MiDAS at telomeres requires RAD52, and is mechanistically similar to CFS-associated MiDAS, with the notable exception that telomeric MiDAS does not require the MUS81-EME1 endonuclease. We propose a model whereby replication stress initiates a RAD52-dependent form of break-induced replication that bypasses a requirement for MUS81-EME1 to complete DNA synthesis in mitosis.

## INTRODUCTION

Telomeres are the specialized DNA structures that cap the ends of linear chromosomes in eukaryotes. Although the sequence of telomeric DNA can differ amongst different organisms, the basic structure is conserved. In humans, each telomere comprises a long dsDNA tract of TTAGGG repeat units that terminates on the G-rich 3′-strand with a ssDNA overhang of variable length [1]. This 3′-overhang is proposed to loop back and invade into the double-stranded telomeric repeat DNA to form a so-called T-loop, which resembles the D-loop generated as an intermediate during homologous recombination [2, 3]. Telomeric DNA in human cells is bound by the shelterin protein complex, which comprises TRF1, TRF2, RAP1, TIN2, TPP1, and POT1. Shelterin proteins serve to maintain the T-loop at telomeres and to ensure that chromosome ends are not mistaken for pathological dsDNA breaks, which ultimately suppresses excessive recombination between telomeres [1]. However, there is an apparent ‘price to pay’ for the unusual organizational structure at telomeres, in that the G-rich repeated sequence, together with its tightly bound shelterin proteins, form a formidable barrier to the smooth progression of DNA replication forks. This type of fork disruption is potentially more problematic within telomeres than elsewhere in the genome because the very end of the chromosome must be replicated unidirectionally from an internal replication origin, and hence a stalled replication fork cannot be rescued through merging with a converging fork [4].

Telomeres display a number of features that are shared with common fragile sites (CFSs). Most notably, telomeres and CFSs are defined as ‘difficult-to-replicate’ loci. In both cases, these loci display structural abnormalities on metaphase chromosomes that are induced either by exposure of cells to the DNA polymerase inhibitor, aphidicolin (APH), or by interfering with the function of the replication stress kinase ATR [5–7]. The manifestation of a problematic replication program at CFSs is their tendency to show gaps/breaks/uncondensed regions (denoted as ‘fragility’) on otherwise fully condensed metaphase chromosomes. In contrast, telomere fragility cannot be observed easily on conventional DAPI-stained metaphases. Instead, the detection of telomere fragility requires the use of fluorescence *in situ* hybridization (FISH) to reveal fragility, which usually takes the form of either multi-telomeric FISH signals or abnormally extended telomeres. The underlying mechanism for chromosome fragility is still debated, and might even differ at different loci. However, evidence has accumulated to suggest that the source of replication fork perturbation might be either the presence of a DNA secondary structure in the template (e.g. a hairpin or G-quartet) or because of a clash between the replisome and the transcription machinery [8].

In somatic cells, telomeres can shorten in length during every round of DNA replication due to the so-called ‘end-replication problem’, which ultimately will limit cell proliferation if not rectified. This shortening necessitates the extension of the telomeric sequence, which can occur via either of two mechanisms. Stem cells and most cancer cells utilize the specialized reverse transcriptase, telomerase, to add additional TTAGGG repeat units to the short telomere using an intrinsic RNA primer [9]. Some cancer cells, however, use a homologous recombination-dependent process called the Alternative Lengthening of Telomeres (ALT) pathway [10]. Recently, the ALT pathway has been shown to involve a DNA repair process called break-induced replication (BIR) that has been characterized in detail only in yeast. This proposed telomeric BIR pathway depends upon the non-catalytic subunit of DNA Polymerase δ, POLD3, which is the human homolog of Pol32 required for BIR in yeast. This BIR-like process is seemingly suppressed in telomerase-positive (henceforth denoted as telomerase+) cells, and therefore is restricted to ALT cells requiring recombination functions for the maintenance of telomere stability [11–13]. In this respect, the ALT pathway also shows similarity to the processes required for maintenance of CFS stability, because BIR has been implicated in the completion of DNA replication at CFSs after the cell has entered the prophase of mitosis. We have shown previously that a RAD52-, MUS81- and POLD3-dependent process termed MiDAS (for Mitotic DNA Synthesis) occurs at CFSs following replication stress [14, 15]. MiDAS at CFSs is unusual for a BIR-like event, in that it apparently does not require RAD51. Indeed, the function of RAD51 appears to suppress a requirement for MiDAS, suggesting that MiDAS might represent an atypical, sub-pathway of BIR, which serves to back-up conventional RAD51-dependent recombination occurring prior to mitosis.

In this study, we report that human cancer cells exhibit MiDAS at telomeres, which is enhanced in response to replication stress (low dose APH). Interestingly, this process is a feature of both ALT cells and telomerase^+^ cells, and is not restricted to telomeres that are overtly fragile. We also show that APH-induced telomeric MiDAS requires a similar, but not identical, set of DNA repair/recombination factors to those that promote CFS-associated MiDAS, highlighting telomeres as a specialized subset of CFSs. Given that oncogene-induced replication stress is a common feature of cancers, we propose that disrupting MiDAS could be a viable strategy to selectively kill malignant cells as it will target both telomerase+ and ALT tumors.

## RESULTS

### Mitotic DNA synthesis (MiDAS) occurs at telomeres

**Figure 1:**
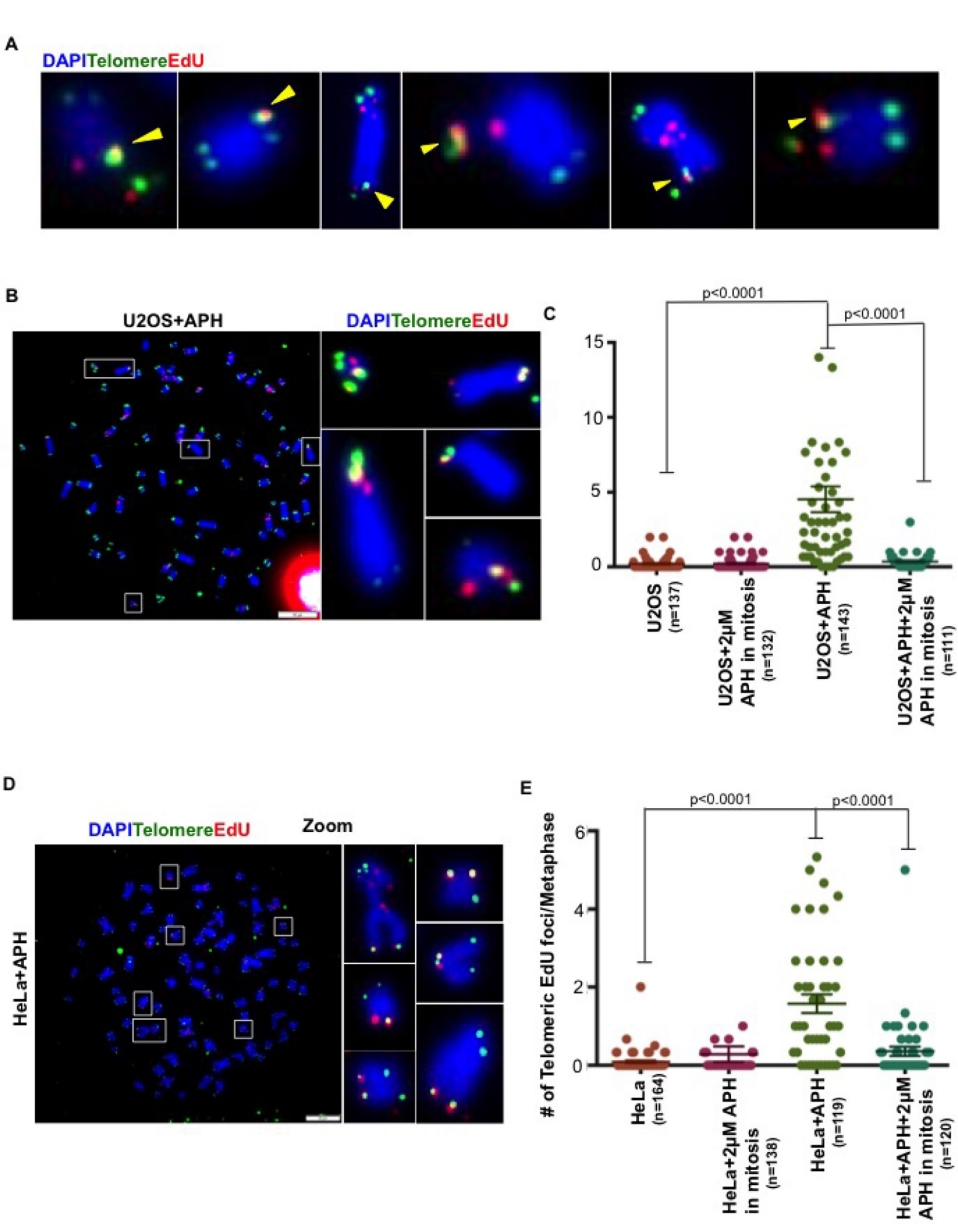
Replication stress induces mitotic DNA synthesis at telomeres in both ALT and telomerase+ cells (**A**) Representative images of EdU (red) incorporation at telomeres (marked by telomeric FISH; green). Yellow arrows show those EdU foci that were scored either because of co-localization with the FISH signal or because the EdU focus lay beyond the tip of the chromosome. (**B**) Representative images of EdU (red) incorporation at telomeres (marked by telomeric FISH; green) on metaphase chromosomes (DAPI stained; blue) in APH-treated U2OS cells. (**C**) Quantification of EdU incorporation at telomeres on metaphase chromosomes in U2OS cells treated with or without low dose APH (0.4 μM) treatment in S phase. Where indicated, cells were exposed to high dose APH (2 μM) in mitosis. (**D**) Representative images of EdU (red) incorporation at telomeres (marked by telomeric FISH; green) on metaphase chromosomes (stained with DAPI; blue) in low dose APH-treated HeLa cells. (**E**) Quantification of EdU incorporation at telomeres on metaphase chromosomes from HeLa cells treated with or without low dose APH (0.4 μM). Where indicated, cells were exposed to high dose APH (2 μM) in mitosis. Data represent the means of at least three independent experiments. Error bars indicate SEM. See also Supplementary Figure 1.

It has been shown previously that cells exposed to APH-induced DNA replication stress conduct BIR-like DNA repair synthesis (MiDAS) at CFS loci in early mitosis [14–16]. Because APH-inducible fragility is also a characteristic of telomeres [7], we investigated whether DNA synthesis might still be occurring within telomeres during mitosis. To this end, we utilized an established EdU labelling method [14, 15] for quantifying MiDAS in U2OS cells that had been treated or not with a low dose of APH (0.4 μM) during S phase. We then analyzed sites of MiDAS on metaphase chromosomes using a combination of telomeric FISH and EdU detection using Click-IT chemistry. To ensure that we omitted examples of MiDAS occurring at CFSs fortuitously located close to a telomere, we only scored those EdU foci that co-localized precisely with a telomeric FISH signal or that lay at the very tip of the chromosome distal to the FISH signal (Figure 1A). Using this approach, we could readily detect EdU incorporation at telomeres during mitosis that was strongly induced by exposure of the cells in S phase to APH (Figure 1B, 1C and Supplementary Figure 1A, 1B). Henceforth, we will refer to this as ‘telomeric MiDAS’. To confirm that this DNA synthesis was dependent upon conventional replicative polymerases, as has been shown for CFS-associated MiDAS, we exposed cells in mitosis to a high dose of APH (2 μM). As expected, telomeric MiDAS was abolished by high dose APH applied in mitosis (Figures 1C and Supplementary Figure 1A, 1B). We conclude that MiDAS occurs frequently at telomeres in U2OS cells undergoing replication stress.

### MiDAS occurs at telomeres maintained by telomerase or ALT

**Figure 2:**
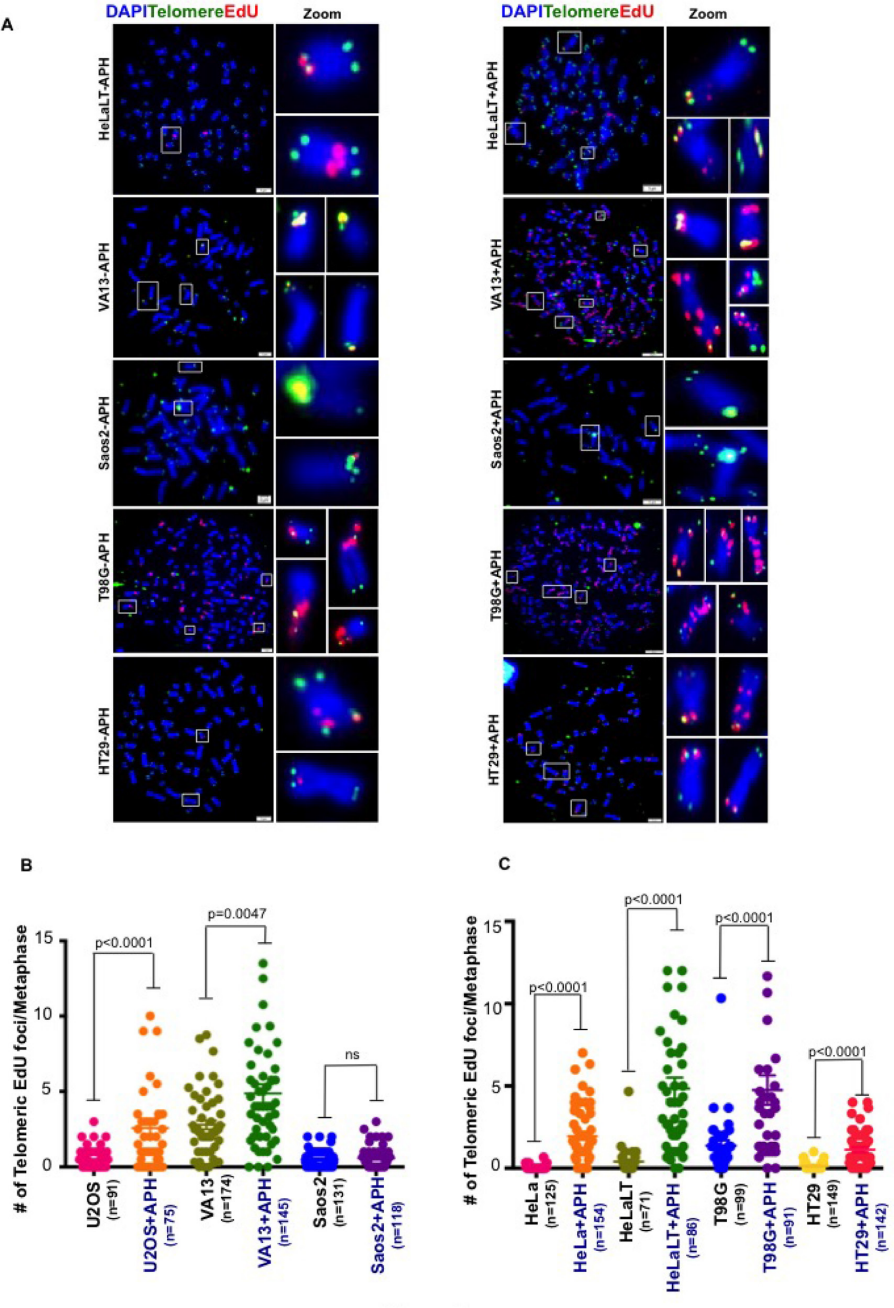
Spontaneous MiDAS at telomeres is not limited to ALT-associated telomeres (**A**) Representative images and quantification of EdU (red) incorporation at telomeres (marked by telomeric FISH; green) on metaphase chromosomes (stained with DAPI; blue) in a series of (**B**) ALT and (**C**) telomerase positive cell lines with and without low dose APH. Data represent the means of at least three independent experiments. Error bars indicate SEM. See also Supplementary Figure 2.

The ALT pathway, like CFS-associated MiDAS, has been proposed to operate via a BIR-like process [11, 13]. Hence, we set out to determine whether telomeric MiDAS was limited to those cells utilizing ALT for their telomere maintenance. For this, we first analyzed HeLa cells, which express telomerase. We observed that replication stress also induced telomeric MiDAS in HeLa cells, which was abrogated by high dose APH treatment in mitosis (Figure 1D, 1E and Supplementary Figure 1C, 1D). These data suggest that APH-induced telomeric MiDAS is not limited to ALT cells, and that the telomerase status of cells does not influence whether replication stress-induced MiDAS occurs at telomeres. To gain additional evidence in support of this contention, we quantified MiDAS in a panel of different ALT and telomerase+ cell lines. These lines also differ in their degree of aneuploidy and/or telomere length. This analysis confirmed that APH-induced telomeric MiDAS occurs not only in ALT cells, but also in cells with telomerase-maintained telomeres (Figure 2A–2C and Supplementary Figure 2). Moreover, in a telomerase+ cell line, T98G, we observed a discernible level of telomeric MiDAS even in the absence of replication stress (Figure 2C). This feature, which is shared with the ALT cell lines, indicates that telomeric MiDAS occurring in the absence of exogenous replicative stress is also not restricted to ALT cell lines. We failed to detect significant levels of APH-induced telomeric MiDAS in only one cell line, Saos-2, which is a near-diploid ALT cell line (Figure 2B). In a sub-clone of HeLa cells with abnormally long telomeres (HeLaLT) [17], we observed a significantly higher level of telomeric MiDAS than was detected in the parental HeLa cells, both in the presence and absence of replication stress (Figure 2C). We conclude that telomeric MiDAS occurs in a wide range of cancer cell lines irrespective of their mechanism of telomere maintenance. Moreover, neoplastic transformation *per se* is not a prerequisite for the existence of replication stress-induced MiDAS, as the VA13 fibroblast cell line also exhibited a robust level of MiDAS (Figure 2B).

### Telomeric MiDAS does not correlate with telomere fragility

**Figure 3:**
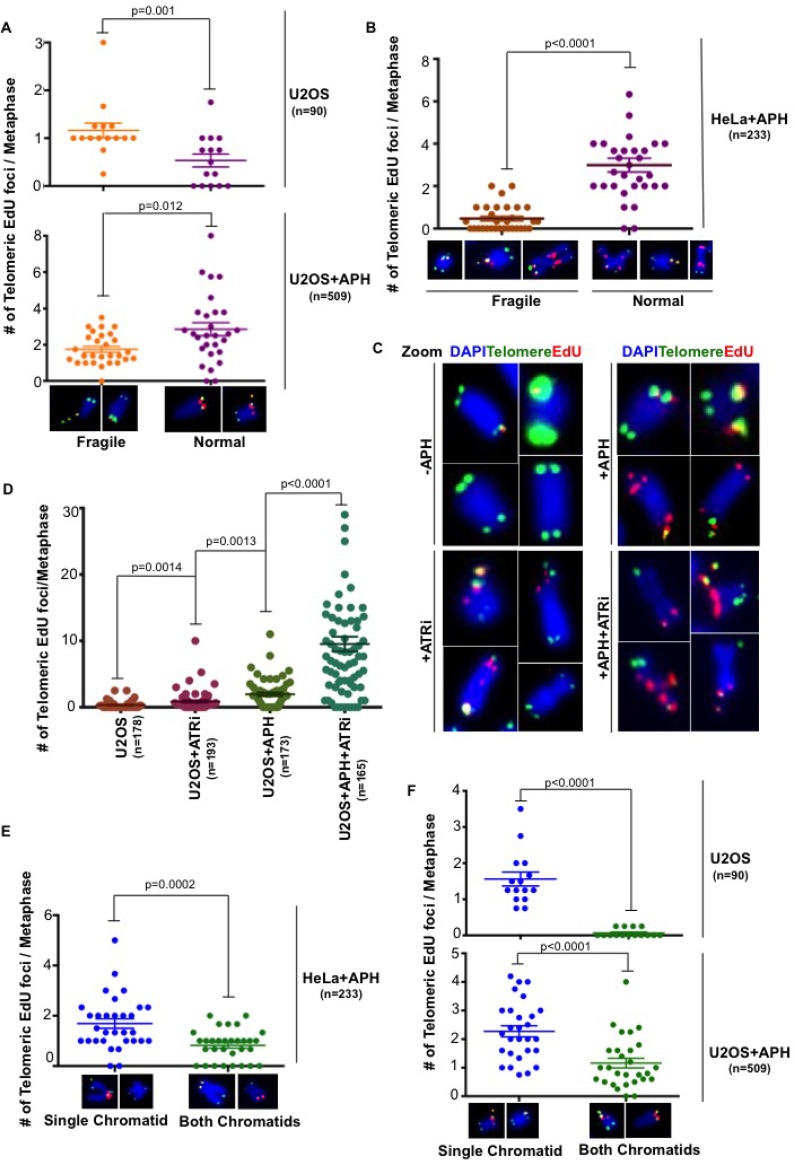
Replication stress induced telomeric MiDAS does not correlated with telomere fragility Quantification of telomeric EdU foci associated with either morphologically fragile or normal telomeres in (**A**) untreated U2OS cells or low dose APH-treated U2OS and (**B**) HeLa cells. (**C**) Representative images and (**D**) quantification of EdU incorporation (red) at telomeres (marked by telomeric FISH; green) on metaphase chromosomes (DAPI stained; blue) in U2OS cells. Where indicated, cells were exposed to low dose APH and 5 μM ATRi. Quantification of telomeric EdU foci located either on a single chromatid or on both chromatids in (**E**) APH-treated HeLa cells and (**F**) U2OS cells in both untreated or APH-treated conditions. Data represent the means of at least three independent experiments. Error bars indicate SEM. See also Supplementary Figure 3.

We showed previously that CFS-associated MiDAS occurs frequently at the site of a visible gap/break in the metaphase chromosome. Hence, we analyzed the relationship between telomeric MiDAS and the presence or absence of overt telomere fragility. In the absence of APH, telomeric MiDAS was largely associated with fragile telomeres in U2OS cells. Nevertheless, despite the apparent similarities between telomere- and CFS-associated MiDAS, there was no association between fragility and the appearance of telomeric MiDAS following APH treatment. On the contrary, telomeric MiDAS induced by APH more frequently occurred at telomeres that exhibited a normal gross morphology in both U2OS and HeLa cells (Figure 3A, 3B). Hence, it is clear that the presence of overt telomere fragility and MiDAS correlate poorly. As expected, considering that telomeric MiDAS is a phenomenon associated with replication stress, the inhibition of the ATR kinase using a small molecule inhibitor, VE-821, exacerbated the level of telomeric MiDAS in U2OS cells (Figure 3C, 3D, and Supplementary Figure 3). This compound has been shown previously to enhance fragility at both telomeres and CFSs [6, 7], and to promote MiDAS at CFSs [14]. We conclude that replication stress-induced telomeric MiDAS differs from CFS-associated MiDAS in that it is not tightly linked to the appearance of fragility.

### MiDAS at telomeres is predominantly a conservative form of DNA synthesis

CFS-associated MiDAS has been shown to occur predominantly via a conservative form of DNA synthesis that leads to EdU incorporation on only one of the two sister chromatids of a mitotic chromosome. Furthermore, the ALT mechanism has been shown to utilize a conservative form of DNA synthesis [13]. To investigate whether telomeric MiDAS was similarly conservative, we quantified the relative proportions of mitotic EdU foci that were present on either a single sister chromatid or both chromatids. We observed that EdU incorporation at telomeres in both U2OS and HeLa cells occurs primarily in a conservative manner irrespective of whether the cells were exposed or not to replication stress (Figure 3E, 3F). We also investigated if there is a correlation between telomere fragility and whether the DNA synthesis occurred via a conservative or a semi-conservative process. However, we failed to observe any correlation in either untreated or APH-treated U2OS cells. For example, in the case of APH-treated cells, 20% of the events were conservative and associated with fragility, while 18% were non-conservative but fragile. For the remaining 62% of events that occurred on non-fragile telomeres, 29% were conservative while 33% were non-conservative.

### Telomeric MiDAS utilizes some, but not all, of the same factors as CFS-MiDAS

**Figure 4:**
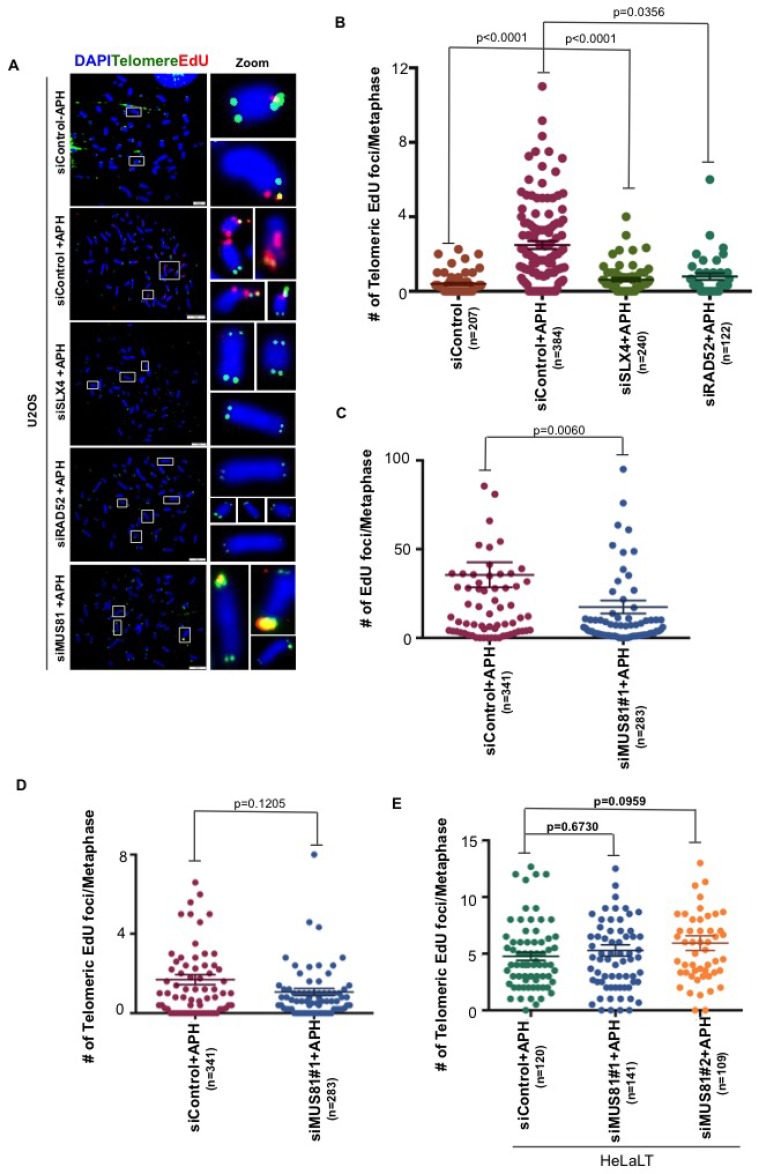
Telomeric MiDAS is also SLX4- and RAD52-dependent, but independent of MUS81 (**A**) Representative images EdU (red) incorporation at telomeres (marked by telomeric FISH; green) on metaphase chromosomes (stained with DAPI; blue) in control, SLX4-, RAD52-, or MUS81-depleted U2OS, as indicated. (**B**–**D**) Quantification of total EdU incorporation (combined telomeric and non-telomeric) and EdU incorporation specifically at telomeres in U2OS cells. (**E**) Quantification of EdU incorporation specifically at telomeres in HeLa-LT cells. EdU was quantified on metaphase chromosomes following the indicated siRNA depletions. Data represent the means of at least three independent experiments. Error bars indicate SEM. See also Supplementary Figure 4.

MiDAS at CFSs is proposed to be a BIR-like process that is mediated by a RAD52-dependent, but RAD51-independent, form of homologous recombination. In addition, CFS-MiDAS has a strong requirement for the SLX4 scaffold protein and its associated endonuclease MUS81-EME1 [14, 15, 18]. To investigate the mechanism for telomeric MiDAS, we chose to focus on U2OS cells due to the high levels of telomeric MiDAS exhibited by these cells. We individually depleted SLX4, RAD52 and MUS81 in U2OS cells. In agreement with previous data on CFSs, we observed that SLX4 and RAD52 are required for telomeric MiDAS (Figure 4A, 4B and Supplementary Figure 4A, 4B). However, in contrast to CFS-associated MiDAS (Figure 4C), the MiDAS occurring at telomeres was apparently independent of MUS81-EME1 in U2OS cells (Figure 4D and Supplementary Figure 4A). To further confirm this in a non-ALT cell line, we chose HeLaLT cells and depleted MUS81 using two previously validated siRNAs. Similarly to U2OS cells, we observed that telomeric MiDAS in HeLaLT cells does not require MUS81 (Figure 4E and Supplementary Figure 4C).

## DISCUSSION

Oncogene-induced DNA replication stress is a key driver of chromosomal instability in human cancer, and is proposed to play a direct role in oncogenesis [19, 20]. It is imperative, therefore, that we understand not only the causes of replication stress, but also the pathways used by cells to counteract its potentially oncogenic effects. When challenged by replication stress, the replication program at CFSs is often disturbed to such an extent that they continue to undertake DNA synthesis even after the cell has entered early mitosis. Recent data indicate that this mitotic DNA synthesis (MiDAS) is not conventional, semi-conservative replication, but rather is a recombinational repair-driven process that resembles BIR in yeast. In this article, we have investigated the role of MiDAS at telomeres. This was undertaken because telomeres resemble CFSs and present a severe challenge to the DNA replication machinery due to their sequence, structure, tightly-bound proteins, and unidirectional replication.

We have shown that MiDAS occurs at telomeres, and is significantly activated by prior APH-induced replication stress. Moreover, our data indicate that telomeric MiDAS and CFS MiDAS can proceed via a RAD52-dependent, conservative form of DNA synthesis, and largely utilize a common set of factors. Nevertheless, there are significant differences between the behaviour of CFSs and telomeres under replication stress. The most notable of these are the lack of a requirement for MUS81 for telomeric MiDAS, and a lack of a correlation between MiDAS and the presence of overt fragility. A form of micro-homology mediated BIR (MMBIR) has been proposed as the mechanism of MiDAS at CFSs, which relies on MUS81-EME1 to promote template switching. There are two potential explanations for the lack of requirement for MUS81 in telomeric MiDAS. First, because telomeres already comprise repetitive sequences, MMBIR-associated template switching might not be required. Second, the telomere-associated 3-way junction generated as an intermediate during BIR might not require to be resolved at all because it could potentially branch migrate until the end of the chromosome. Although our data are consistent with the proposal that telomeres represent *bona fide* fragile sites in the human genome, we observed a tendency for APH-induced MiDAS to occur at telomeres with a morphologically normal appearance. This was the case for both U2OS cells (ALT) and HeLa cells (telomerase+). Hence, it is clear that the presence of telomeric MiDAS and fragility correlate poorly, and should not be viewed as surrogates of one another.

Recently, a form of POLD3-dependent DNA synthesis was reported to occur at telomeres in the absence of replication stress, which was termed break-induced telomere synthesis. This process resembles the telomeric MiDAS that we report here, but with one crucial difference: the telomeric DNA synthesis was observed only in ALT cells, and not in telomerase+ cells, and was therefore considered to be the mechanism by which ALT occurs [11]. In contrast, we consistently observed telomeric MiDAS in both telomerase+ and ALT cell lines. Therefore, telomeric MiDAS is not specific for cells with ALT-mediated telomere maintenance, but instead appears to be a general feature influenced by numerous telomere-associated features, including telomere length, as well as the extent of aneuploidy in cells. These features would potentially create a challenge to the faithful replication of a telomere and increase the probability that fork stalling and associated DNA damage would occur during S phase. Taken together with the previously published results, our data underline how both intrinsic factors (telomere length and aneuploidy) and extrinsic factors (APH-induced replication stress and DSB induction), but not ALT *per se*, are the main drivers of MiDAS. Interestingly, given the association of replication stress with oncogenesis, the existence of high levels of telomeric (and CFS-associated) MiDAS levels in the SV40-transformed VA13 fibroblast line indicates that neoplastic transformation is not a prerequisite for the existence of the MiDAS pathway.

Recently, Min *et al*. [11, 21] reported a telomeric MiDAS pathway that was proposed to underlie the ALT mechanism. These authors showed that ALT-associated MiDAS occurring in the absence of replication stress is a RAD51- and BRCA2-independent pathway that utilizes RAD52. Moreover, these authors showed that the G-quadruplex stabilizing agent, pyridostatin, induces telomeric MiDAS in both ALT and telomerase+ cells, presumably through stabilizing G-quadruplexes within telomeric DNA that then generates localized replication stress. These data on ALT cells correspond with ours, but we additionally have shown that this pathway can be responsible for high levels of basal (as well as APH-induced) telomeric MiDAS in certain telomerase+ cells. In addition, Min *et al*. concluded that basal telomeric MiDAS is ATR-dependent, while we find (as for CFS-associated MiDAS) that ATR suppresses telomeric MiDAS both untreated and APH-treated cells. The reasons for these discrepancies are not clear at this stage. Indeed, we propose that aneuploidy is a key driver of MiDAS both at telomeres and CFSs, which further supports the notion that the use of MiDAS inhibitors could be a promising strategy for anti-cancer therapy [14].

## MATERIALS AND METHODS

### Cell culture

U2OS, VA13, and Saos2 were used as examples of ALT positive cell lines, and HeLa, HeLaLT, HT29, and T98G were used as examples telomerase positive cell lines. U2OS, HeLa, and HT29 cells were obtained from the ATCC. Saos2, VA13, T98G cells were provided by Dr. J. Pena Diaz (University of Copenhagen, Denmark), and HeLaLT cells were provided by Dr. J. Karlseder (Salt Institute, USA). All cell lines were maintained in Dulbecco’s modified Eagle’s medium (GlutaMAX; Thermo Scientific) supplemented with 10% fetal bovine serum (Life Technologies) and 1% penicillin/streptomycin (Thermo Scientific) at 37° C in a humidified atmosphere with 5% CO_2_. The cells were screened regularly for mycoplasma using a MycoAlert Mycoplasma Detection Kit (Lonza), and found to be negative.

### Cell synchronization and treatment

For the synchronization and aphidicolin treatment of the cells, a protocol described previously [14, 15] was used with minor modifications. Briefly, the cells were treated with or without 0.4 μM APH (Sigma-Aldrich) for 16 hrs. Cells were synchronized in the late G2 phase with the CDK1 inhibitor RO3306 (7 μM; APExBIO, [22]) either simultaneously or during the last 8 hrs of the APH treatment. The cells were then released from this arrest by washing 3× with PBS at room temperature, and were then allowed to progress into metaphase at 37° C for 45–60 minutes in the presence of 20 μM EdU (Life Technologies) and 100 ng/ml KaryoMAX Colcemid Solution in HBBS (Life Technologies). Where stated, in addition to EdU and Colcemid, the cells were also released into medium containing high dose APH (2 μM). To prepare metaphase chromosome spreads, mitotic cells were shaken off and collected by centrifugation.

Where required, ATR activity was inhibited using 5 μM VE-821 (SML1415; Sigma-Aldrich) for 16 hrs [14, 23]. VE-821 was added at the same time as the low dose APH, but was kept in the medium after release of cells into mitosis.

### RNA interference

The following siRNAs were used: SLX4 (5′-GAGAAGAACCCUAAUGAAA-3′ and 5′-GCACAA GGGCCCAGAACAA-3′; Sigma Aldrich, published previously in [24], RAD52 (5′-GGUCAUCGGGUAAU UAAUC-3′; J-011760-06-0010, Dharmacon), MUS81 (#1: 5′-CCUAAUGGUCACCACUUCUUA-3′ and #2: 5′-GAGUUGGUACUGGAUCACAUU-3′; Sigma Aldrich, previously published in [25]), and non-targeting siRNA (D-001810-10-20; Dharmacon ON-TARGETplus pool). Cells were transfected with the indicated siRNAs using Lipofectamine RNAimax transfection reagent (Life Technologies).

### Metaphase spreads

Cells arrested in G2 by exposure to 7 μM RO3306 (APExBIO; [22]) were released into mitosis in the presence of 100 ng/ml KaryoMAX Colcemid Solution in HBBS (Life Technologies). Mitotic cells were collected and harvested by centrifugation at 300 g for 5 minutes. The cells were swollen with pre-warmed 37.5 mM KCl at 37° C for 20–30 minutes, and were then harvested at 250 g for 5 minutes. Ice-cold methanol:acetic acid (3:1) was used to fix the cells at −20° C for overnight. Fixed cells were re-suspended in fresh fixative and dropped onto pre-cooled damp slides to make chromosome spreads. The slides were aged either at room temperature for a week, at 42° C overnight or at 65° C for 10 minutes before Telomeric FISH analysis and/or EdU detection (see below).

### Telomeric FISH

A FAM-labeled, C-rich PNA telomere probe (Panagene) was used for telomeric FISH according to the manufacturer’s protocol with the following modifications: 1X blocking reagent (11096176001, Roche) was added into the hybridization buffer, probe incubation was performed at 37° C for at least 2 hours, and the slides were incubated in Washing Solution I (PBS with 0.1% Tween 20, pre-warmed at 57° C) for 3 × 10 min. Following the FISH analysis, EdU detection (see below) was performed after re-fixation with 4% formaldehyde in PBS for 4 minutes at room temperature.

### EdU labeling and detection

Cells were pulse labeled with 20 μM EdU (Life Technologies) for 45–60 minutes after release from an RO3306 arrest. Metaphase chromosomes were isolated (see above) and EdU detection was performed with a Click-IT EdU Alexa Flour 594 Imaging kit (Life Technologies), as described previously [14].

### Microscopy analysis

Images were captured with an Olympus BX63 microscope and the images were then analyzed using Olympus CellSens software.

### Antibodies

The primary antibodies and their dilutions for Western blotting analysis were RAD52 (1:100 Santa Cruz sc-8350, or 1:500 Abcam ab103067), SLX4 (1:200, S714C raised against the residues 1535-1834, a gift from Dr. J. Rouse, University of Dundee, UK), MUS81 (1:1000, Santa Cruz sc-53382), histone H3 (1:2000, Abcam ab1791), actin (1:1000, Sigma-Aldrich A3853), and β-tubulin (1:1000, T4026; Sigma Aldrich). The secondary antibodies used were goat anti-rabbit IgG (1:5000, Sigma-Aldrich A6667), goat anti-mouse IgG (1:5000, Sigma Aldrich A4416), and donkey anti-sheep IgG (1:5000, Sigma Aldrich A3415).

### Western blotting analysis

The cells were lysed with high-salt RIPA buffer (50 mM Tris-HCl, pH7.5, 420 mM NaCl, 1.0% NP-40, 0.5% deoxycholate, 0.1% SDS, 10% glycerol) containing protease inhibitor cocktail (cOmplete EDTA-free protease inhibitor cocktail tablets; Roche) for 30 minutes on ice. A BCA protein assay kit (Thermo Fisher Scientific) was used to measure protein concentration according to the manufacturer’s instructions. The protein samples were run on 4–12% or 12% NuPAGE Bis-Tris gels (Life Technologies) with NuPAGE MOPS SDS running buffer (Life Technologies), and were transferred to a PVDF membrane (Amersham Hybond PVDF membrane; VWR) using wet transfer. The primary antibody incubation was performed for 16 hours at 4° C following blocking of the membrane in 5% milk in PBS-T (0.1% Tween 20 in PBS) for 1 hour at room temperature. After washing in PBS-T, the membrane was incubated with the secondary antibody for 30 minutes at room temperature. The membrane was washed and incubated with Luminata Forte Western HRP Substrate (Millipore) for signal detection. The western blot images were captured using an Amersham Imager 600 and quantified with Fiji software (GE Life Sciences).

### Statistical analysis

Statistical significance was calculated using a Mann-Whitney test for unpaired and nonparametric data with GraphPad Prism Software (version 6).

## SUPPLEMENTARY MATERIALS FIGURES AND TABLES


