# Supplementary material for: Human cancer cells utilize mitotic DNA synthesis to resist replication stress at telomeres regardless of their telomere maintenance mechanism
Source: Oncotarget. 2018 Mar 23;9(22):15836–46. doi: 10.18632/oncotarget.24745 (PMC5882301; doi:10.18632/oncotarget.24745)
Supplement: Supplementary file 1 [file oncotarget-09-15836-s001.pdf]

Human cancer cells utilize mitotic DNA synthesis to resist replication stress at telomeres regardless of their telomere maintenance mechanism

SUPPLEMENTARY MATERIALS

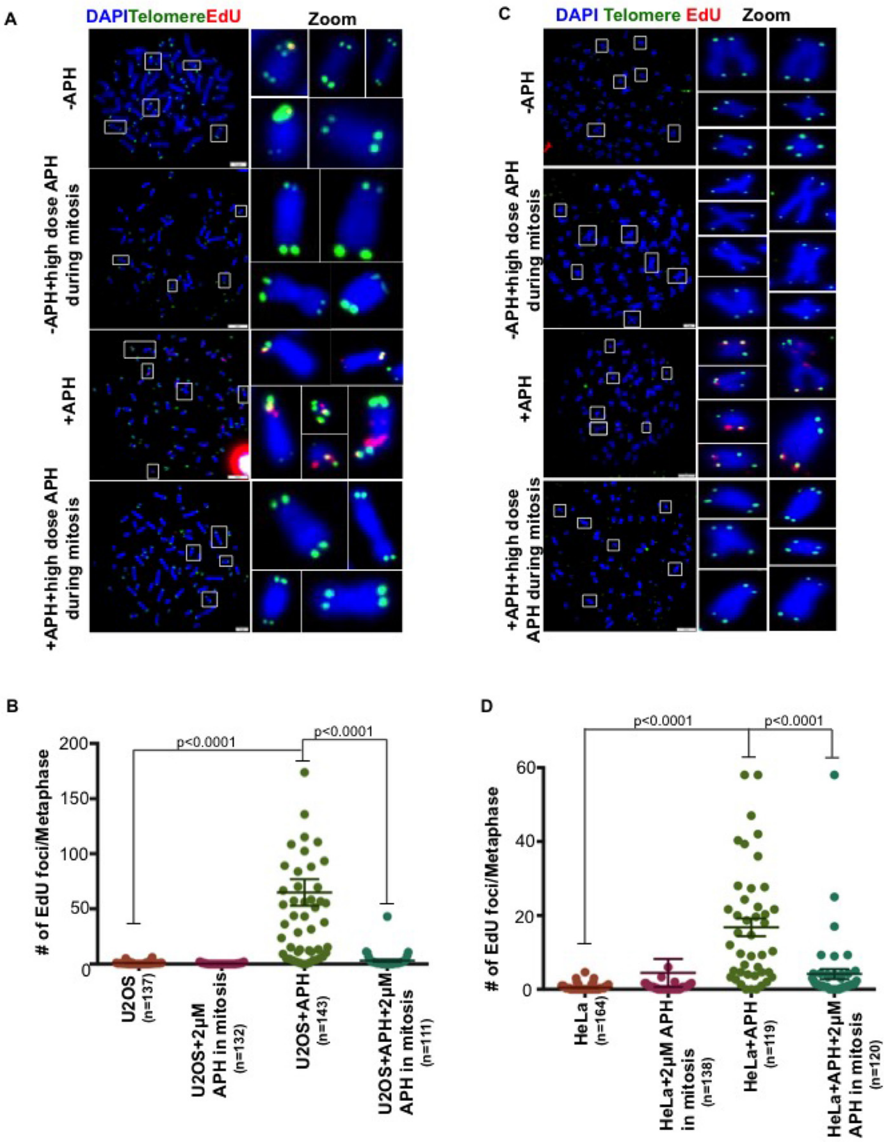

Supplementary Figure 1: Related to Figure 1. (**A**) Expanded representative images of EdU (red) incorporation at telomeres (marked by telomeric FISH; green) on metaphase chromosomes (stained with DAPI; blue) in U2OS cells treated with or without low dose APH (0.4 μM). Where indicated, cells were exposed to high dose APH (2 μM) in mitosis. (**B**) Quantification of total EdU incorporation on all loci of metaphase chromosomes in U2OS cells treated with or without low dose APH (0.4 μM). Where indicated, cells were exposed to high dose APH (2 μM) in mitosis. Connected to Figure 1C. (**C**) Expanded representative images of EdU (red) incorporation at telomeres (marked by telomeric FISH; green) on metaphase chromosomes (stained with DAPI; blue) in HeLa cells treated with or without low dose APH (0.4 μM). Where indicated, cells were exposed to high dose APH (2 μM) in mitosis. (**D**) Quantification of the total EdU incorporation on metaphase chromosomes from HeLa cells treated with or without low dose APH (0.4 μM). Where indicated, cells were exposed to high dose APH (2 μM) in mitosis. Connected to Figure 1E. Data represent the means of at least three independent experiments. Error bars indicate SEM.

A

	#Chromosomes	#Telomere Maintenance	Origin
HeLa	82, 70-164	Telomerase +	cervix, adenocarcinoma
HeLaLT	aneuploid & long telomeres	Telomerase +	derived from HeLa 1.2.11, telomeres up to 40kb
HT29	71, 68-72	Telomerase +	colon, colorectal adenocarcinoma
T98G	128-132	Telomerase +	brain, glioblastoma multiforme
U2OS	aneuploid	ALT	bone, osteosarcoma
VA13	76, 45-89	ALT	lung, SV40
Saos2	2n=46	ALT	bone, osteosarcoma

B

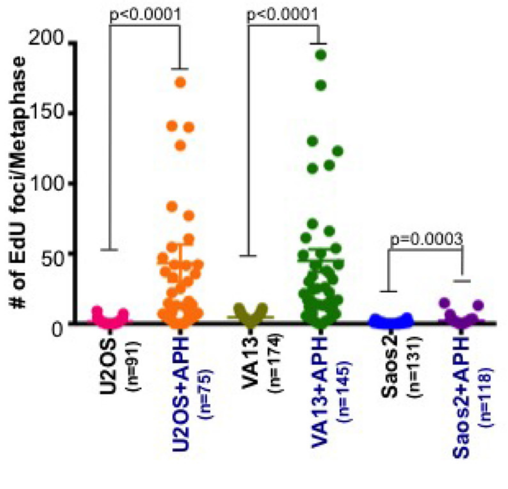

C

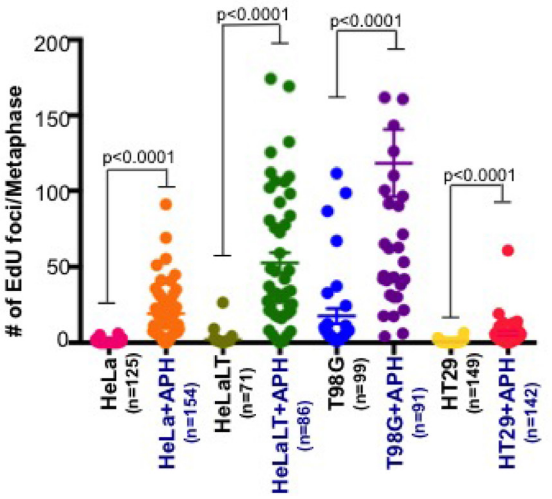

Supplementary Figure 2: Related to Figure 2. (**A**) The chromosome number, telomere maintenance mechanism, and origin information of the range of ALT and telomerase + cell lines used. Quantification of the total EdU incorporation at telomeres on metaphase chromosomes in a series of (**B**) ALT and (**C**) telomerase + cell lines with or without APH. Connected to Figures 2B and 2C. Data represent the means of at least three independent experiments. Error bars indicate SEM.

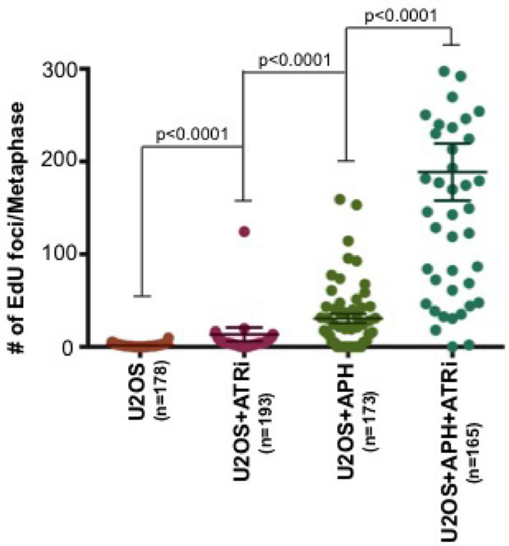

Supplementary Figure 3: Related to Figure 3. Quantification of the total EdU incorporation at telomeres on metaphase chromosomes in U2OS with or without low dose APH. Where indicated, cells were exposed to 5 µM ATRi. Connected to Figure 3D. Data represent the means of at least three independent experiments. Error bars indicate SEM.

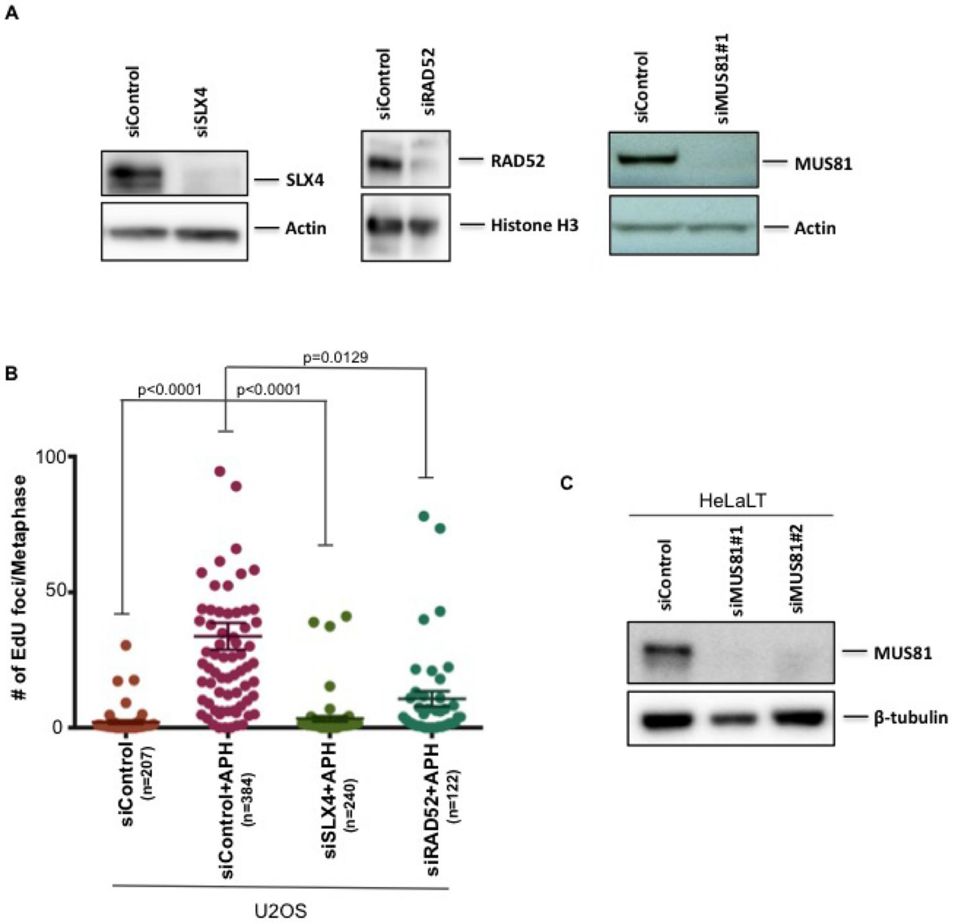

Supplementary Figure 4: Related to Figure 4. (**A**) Representative western blot images of U2OS cells following the indicated siRNA depletions. (**B**) Quantification of total EdU incorporation on metaphase chromosomes in control, SLX4- and RAD52-depleted U2OS cells treated with low dose APH. (**C**) Representative western blot images of HeLaLT cells following siRNA-mediated depletion of MUS81. Connected to Figure 4. Data represent the means of at least three independent experiments. Error bars indicate SEM.
